# Impact of Pressure Guidewire on Model-Based FFR Prediction

**DOI:** 10.1007/s13239-024-00710-9

**Published:** 2024-03-04

**Authors:** Alessia Lucca, Luigi Fraccarollo, Fredrik E. Fossan, Anders T. Bråten, Silvia Pozzi, Christian Vergara, Lucas O. Müller

**Affiliations:** 1 Department of Mathematics, University of Trento, Via Sommarive, 14, 38123 Trento, Italy; 2 DICAM, University of Trento, Trento, Italy; 3 Department of Structural Engineering, Norwegian University of Science and Technology, Trondheim, Norway; 4 Department of Cardiology, Norwegian University of Science and Technology, Trondheim, Norway; 5 MOX, Dipartimento di Matematica, Politecnico di Milano, Milan, Italy; 6 LABS, Dipartimento di Chimica, Materiali e Ingegneria Chimica ”Giulio Natta”, Politecnico di Milano, Milan, Italy

**Keywords:** CAD, FFR, Pressure guidewire

## Abstract

**Introduction:**

Fractional Flow Reserve (FFR) is used to characterize the functional significance of coronary artery stenoses. FFR is assessed under hyperemic conditions by invasive measurements of trans-stenotic pressure thanks to the insertion of a pressure guidewire across the coronary stenosis during catheterization. In order to overcome the potential risk related to the invasive procedure and to reduce the associated high costs, three-dimensional blood flow simulations that incorporate clinical imaging and patient-specific characteristics have been proposed.

**Purpose:**

Most CCTA-derived FFR models neglect the potential influence of the guidewire on computed flow and pressure. Here we aim to quantify the impact of taking into account the presence of the guidewire in model-based FFR prediction.

**Methods:**

We adopt a CCTA-derived FFR model and perform simulations with and without the guidewire for 18 patients with suspected stable CAD.

**Results:**

Presented results show that the presence of the guidewire leads to a tendency to predict a lower FFR value. The FFR reduction is prominent in cases of severe stenoses, while the influence of the guidewire is less pronounced in cases of moderate stenoses.

**Conclusion:**

From a clinical decision-making point of view, including of the pressure guidewire is potentially relevant only for intermediate stenosis cases.

## Introduction

Coronary artery disease (CAD) is one of the leading causes of death in the world [1]. CAD is caused by the buildup of atherosclerotic plaques in coronary vessel wall, resulting in a reduction in oxygen supply to heart tissue and possibly leading to cardiovascular-related events such as myocardial infarction and unstable angina [2].

In clinical setting, both medical imaging techniques and invasive functional assessment procedures are used for the detection of stenoses in coronary arteries and, depending on the characteristics of the atherosclerotic lesions, several alternative treatments are applied [3]. Currently, the gold standard for diagnosis of functional severity of ischemia-inducible coronary stenosis is the Fractional Flow Reserve (FFR) [4]. Clinically, FFR of a given coronary lesion is assessed invasively after administration of a pharmacologial vasodilator agent (i.e. adenosine, papaverine) to induce hyperemia. During transfemoral or transradial catheterization a guiding wire equipped with a miniaturised pressure sensor is inserted into the coronary artery to record simultaneously the pressure in the aorta ($p_{a}$) and the pressure approximately 2–3 cm distal to the lesion ($p_{d}$) that is to be investigated. FFR is then determinate as a ratio of the mean of $p_{d}$ and $p_{a}$ tracings [5]. FFR thresholds are defined in order to guide therapy for stable CAD and to decide whether a surgical procedure is needed or patients can just be treated with optimal medical therapy. Trials evaluating the prognostic impact of the FFR have shown that revascularisation can be safely deferred if FFR is $>0.80$, while a lesion is haemodynamically relevant if FFR $<0.75$ and then revascularisation is recommended [5]. There exists a gray zone for FFR between 0.75 and 0.8, where sound clinical judgment should balance the final decision. Even though the European Society of Cardiologists recommends the use of FFR to guide therapy for stable CAD [6], FFR remains underused due to associated costs, its invasive nature and the need of trained interventionalists. This underuse has lead the medical community to look into non-invasive screening tools to select patients that will likely have functional significant lesions.

Coronary computed tomography angiography (CCTA) has emerged as a non-invasive method to identify geometrical significance of a lesion. Studies have shown that it is characterized by high sensitivity and low specificity. Consequently, among CCTA-identified stenoses, only a minority are then found to be functional significant (FFR < 0.8) [7]. Image-based modelling in combination with computational fluid dynamics has proved to be an effective answer to the need of a more selective non-invasive method. This approach allows to predict FFR using only CCTA scans and non-invasive subject-specific clinical data. Fully physics-based models, relying on solving the incompressible Navier–Stokes equations in complex, three-dimensional domains, and also reduced-order models, based on one-dimensional blood flow equations and one-dimensional image processing without the use of supercomputers, have shown a high diagnostic performance [8–10]. CCTA-derived FFR has proved to complement the anatomical information provided by CCTA to aid diagnosis and reduce the number of unnecessary invasive procedures conducted in patients who turn out to have non-flow-limiting coronary artery stenosis.

Although the presence of the guidewire is often neglected, studies conducted both in vitro [11] and computationally with idealized geometries and in a patient-specific domain [12] have shown that the haemodynamic alteration caused by the presence of the guidewire, can lead to an underestimation of the FFR predicted by the clinical invasive measure.

The goal of this article is to quantify the impact of considering the presence of the pressure guidewire in FFR prediction for a wide range of FFR values and considering several patients. To this end, we adopted the CCTA-derived FFR model proposed and validated in [8] and [13] and we introduced a new modelling feature which accounts the presence of the pressure guidewire used in clinical context of CAD detection. To assess its impact we performed three-dimensional computer simulations in the configuration with and without the presence of the pressure guidewire on a sample of 18 patients with suspected stable CAD. Flow rates, pressure distributions and predicted FFR in both configurations were then analyzed and compared.

## Materials and Methods

### Patient Population

We consider a population of 18 patients recruited as part of a clinical trial at St. Olavs hospital, Trondheim, Norway [14]. The subjects included in this study had undergone invasive angiography with FFR measurements after CCTA recommendations. The exclusion criteria applied during the recruitment phase were the following: unstable coronary artery disease; previous percutaneous coronary intervention or bypass surgery; renal insufficiency; contraindication to use of vasodilator agents and non diagnostic quality of the CCTA.

The patients presented at least one suspected lesion, resulting in a collection of 24 FFR measurements. Patients were randomly selected from a larger patient pool in order to obtain a homogeneous distribution of invasive FFR values among four different ranges of values: 0.38$-$0.52, 0.52$-$0.72, 0.72$-$0.84, 0.84-1. In this way, each defined range comprises six FFR measurements. Tables 1 and 2 provide an overview of general patient characteristics and invasive FFR measurements.

**Table 1:** Patient-specific data for the 18 patients considered in this work

Patient IDs	MAP (mmHg)	CO (L min^-1^)	Dominance
1	93.33	6.0	Right
2	95.67	3.8	Right
3	92.67	6.2	Right
4	97.67	6.5	Right
5	84.33	4.4	Right
6	99.33	5.2	Right
7	95.33	3.6	Right
8	100.33	6.3	Left
9	98.67	3.4	Right
10	100.67	5.4	Left
11	115.33	6.4	Right
12	92.33	4.9	Right
13	88.67	6	Right
14	99.33	3.97	Right
15	90.0	4.3	Right
16	105.33	4.66	Right
17	99.0	3.88	Right
18	100.0	5.25	Right

Right dominant coronary circulation means that the posterior descending artery is supplied by the right coronary artery, while the coronary circulation is left dominant if the posterior descending artery is supplied by the left circumflex artery. Hence, the coronary arterial dominance influences which portions and regions of the myocardium is perfused by the right or left coronary branch/circulation and have an impact on the amount of flow entering a particular branch (left or right). MAP (mean aortic pressure), CO (cardiac output) and coronary arterial dominance are reported

**Table 2:** Data for invasive FFR measurements

FFR IDs	Patient IDs	Lesion location	FFR
1	1	mLAD	0.68
2	2	mLAD	0.52
3	2	dLAD	0.46
4	2	pLCX	0.88
5	3	mLAD	0.87
6	4	pLAD	0.5
7	4	2^nd^ diagonalLAD	0.51
8	5	LCX	0.71
9	6	pLAD	0.6
10	7	mLAD	0.59
11	8	LCX	0.38
12	9	mLAD	0.92
13	10	pRCA	0.74
14	11	pLAD	0.7
15	12	mLAD	0.8
16	13	mLAD	0.77
17	13	LCX	0.72
18	14	mRCA	0.96
19	15	mLAD	0.44
20	16	mLAD	0.78
21	16	LCX	0.52
22	17	mLAD	0.83
23	17	1^st^ diagonalLAD	0.89
24	18	dRCA	0.84

Location of the lesion and FFR clinically measured are reported. Prefixes p, m, d represent the proximal, the mid and the distal tract of the coronary artery to which they are related. Nomenclature according to [15]. LAD: left anterior descending artery; LCX: left circumflex artery; RCA: right coronary artery

### Data Collection and Processing

#### Medical Data Acquisition

CCTA was performed using two CT scanners with $2\times 128$ detector rows (Siemens dual source Definition Flash) following a standardized protocol.

FFR was measured using Verrata Plus (Philips Volcano, San Diego, USA) pressure wires according to standard practice. Prior to inserting the pressure wire into the coronary artery, intracoronary nitroglycerin was administered and hyperemia was induced by continuous intravenous infusion of adenosine. Pressure and ratio $p_{d}/p_{a}$ were recorded over several cycles and FFR was taken equal to the lowest observed value of the ratio.

Standard non-invasive diastolic and systolic pressure measurements were performed on both arms as a part of clinical routine, while cardiac output (CO) was calculated on the basis of the cross-sectional area of the left ventricle outflow tract and velocity time integral derived from Pulse Wave Doppler.

#### Coronary Vessel Segmentation and Volume Meshing

Starting from the CCTA scan, the geometry representing the vascular lumen of the coronary tracts of interest was segmented using the open-source software ITK-SNAP [16]. Surface mesh processing, addition of flow extensions and 3D meshing was performed using the open-source library Vascular Modeling ToolKit [17], leading to the anatomical model.

**Fig. 1:**
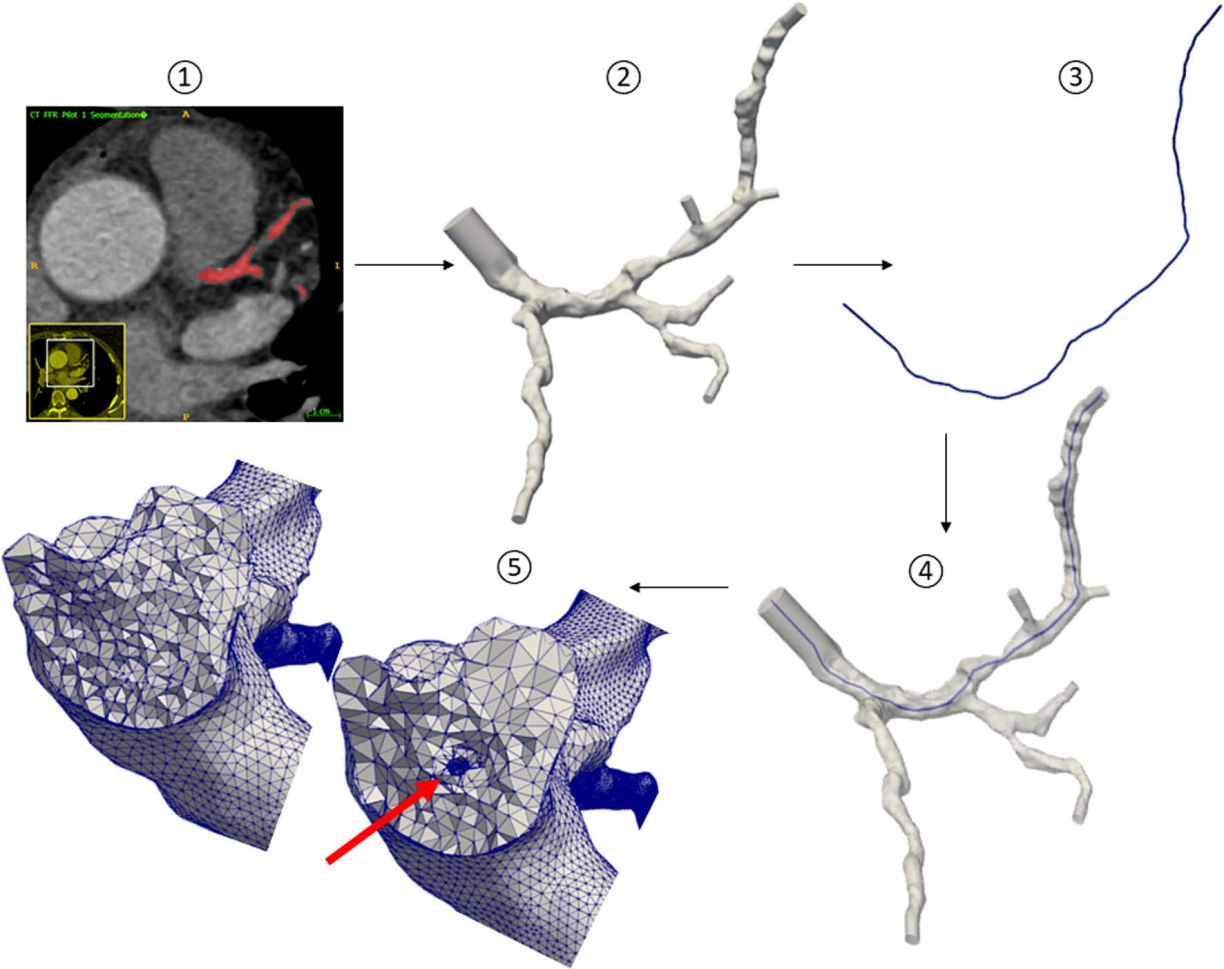
Illustration of the segmentation and meshing process: (1) coronary artery segmentation, (2) preparation of computational domain, (3) guidewire creating from stenotic branch centerline, (4) Boolean difference between the mere coronary tree and the tube, (5) sections of both meshes: with and without the guidewire. The red arrow highlights the region where the pressure guidewire is located

To investigate the influence of the physical presence of a pressure wire in the clinical measurements of FFR, it is necessary to reproduce the fluid dynamic situation also in its presence. The guidewire was modelled as a curvilinear tube of given diameter (0.036 cm, according to the diameter of the pressure guidewire used in the clinical setting), created starting from the centerline of the stenotic branch. Its presence in the stenotic branch was reproduced by performing a Boolean difference between the mere anatomical model and the tube representing the catheter inserted axially into the stenotic vessel, using the software Blender [18]. The last step involved the meshing process performed using Gmsh software [19]. A schematic representation of the meshing process for the insertion of the pressure guidewire into the stenotic branch is reported in Fig. 1.

### Computational Model for Blood Dynamic

Although some works considered compliant vessels for numerical simulations in stenotic coronaries (see e.g. [20, 21]), in this work the segmented coronary tree was considered as rigid domain, whose boundary was decomposed into the inlet section, the surface delimiting the vessel lumen jointly with the interface between the surface of the wire and the blood and N outlets sections, depending on the patient. We assumed the flow regime to be laminar and the blood was modelled as a homogeneous incompressible Newtonian fluid with density 1.05 g cm^-3^ and viscosity 0.035 g cm^-1^ s^-1^. Then, to describe its fluid dynamic behaviour Navier–Stokes equations for incompressible flows supplemented with the initial status of the fluid velocity and with boundary conditions were discretize both in space and time and were numerically solved. The numerical scheme applied is the Incremental Pressure Correction Scheme, described in [22] together with a backward Euler method for time discretizartion. The time step was set to $\Delta t = 1$ms.

**Fig. 2:**
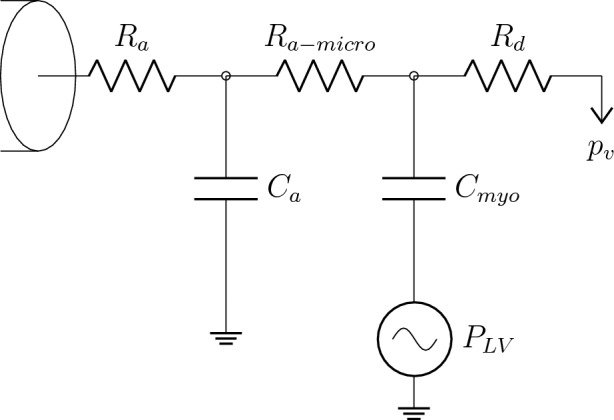
Schematic representation of the coronary bed model coupled to each domain outlet. The coronary bed is embodied by a single arterial path ($R_{a}$, $C_{a}$, $R_{a-micro}$) and a single venous path represented only by a distal resistance $R_{d}$. The two paths are connected by the myocardial compliance $C_{myo}$ which is affected by the time-varying left ventricular pressure $P_{LV}$ [8]. $R_{a}$ represents the coronary arterial resistance, $C_{a}$ denotes the coronary arterial compliance and $R_{a-micro}$ identifies the coronary arterial micro-circulation resistance

In this work we performed simulations considering two different settings, adopting the methodology described in Fossan et al. [8] and Müller et al. [13]. We imposed pressure as inlet boundary condition and, in the first setting (Scenario I), flows at all outlets via prescribed parabolic velocity profile, while in the second setting (Scenario II) we coupled each outlet to a lumped parameter model. The lumped parameter setup, depicted in Fig. 2, is derived from the original work by Mantero et al. [23] and represents a Windkessel model composed of three resistances and two compliances, one of which is connected to a pressure source. Physiologically, the driving factor which primarily accounts for the perfusion of the myocardium is the intramyocardial pressure. More complex models, as that adopted in the work of Mynard et al. [24], incorporate the intramyocardial pressure directly in the lumped parameter model. In such models, peripheral resistance is a function of the compartment volume, which in turn is affected by intramyocardial pressure. The model exploited in this study was chosen based on its ability to represent typical coronary flow patterns with diastolic dominant flow (low systolic flow and peak flow in early diastole) with a simpler setting. An external pressure which in this case is represented by the left ventricle pressure, is applied to capacitors, mimicking the compression of peripheral vessels inside the myocardium. This coupling ensures physiological boundary conditions which take into account the presence of the remaining part of the circulatory system and also the increased impedance experienced by the coronary arteries during the systole. In addition, in both settings a no-slip condition was imposed at the lumen surface and on the interface between the guidewire surface and the blood.

The simulations were performed with FEniCS using CBCFLOW [25]. The computational meshes are composed of tetrahedral elements. The average ratio between a tetrahedral edge length and the radius of the vessel at a given location was set to 0.21 for the wire-absent configuration and to 0.18 for the wire-included configuration resulting in meshes that have on average 985181 elements, (see Fig. 1). The discretization is based on finite element methods. It has been used piecewise-quadratic polynomials to approximate the velocity field, while linear polynomials were used for the pressure. A mesh independence study was performed on two geometries with and without guidewire to verify that the adopted meshing parameter, which in turn defines the mesh elements density, resulted in mesh independent solutions in terms of FFR prediction (relative error respect to solution obtained with the finest mesh below to $1e-2$). See [8] for further details related to the 3D framework.

### FFR Prediction Modelling Pipeline

The approach for non-invasive FFR prediction presented in this study follows the modelling pipeline first introduced by Müller et al. [13]. We computed a baseline state using clinical data according to Scenario I, then based on the resulting distribution of pressure and flow, a hyperemic state was predicted under Scenario II.

The modelling pipeline consists of the following steps:

1. define total baseline flow that enters the coronary tree;
2. distribute the flow among the *N* outlets according to one among several flow distribution criteria available in the literature [13];
3. perform a baseline steady state simulation with prescribed inlet pressure and prescribed outlet flows (Scenario I);
4. compute baseline peripheral coronary resistances according to the pressure and flow distribution of the resting simulation
  1 $$\begin{aligned} R_{out,k}^{bln}=\frac{p_{out,k}^{bln}-p_v}{q_{out,k}^{bln}}\,\, k=1,\dots ,N \end{aligned}$$
  where $p_{out,k}^{bln}$ and $q_{out,k}^{bln}$ are the pressure and flow at $k-th$ outlet resulted from the baseline simulation performed in Step 3 and $p_v$ is a reference venous pressure set to $p_v= 5$ mmHg;
5. compute hyperemic peripheral coronary resistances
  2 $$\begin{aligned} R_{out,k}^{hyp}=\frac{R_{out,k}^{bln}}{TCRI}\,\, k=1,\dots ,N \end{aligned}$$
  where Total Coronary Resistance Index (TCRI) is a hyperemic factor to account for the effect of drug on peripheral coronary arteries vasodilation required to clinically measure FFR. The pipeline adopted assumes TCRI to be $TCRI=3$ which results in coronary flow reserve values close to those measured on average on a population of patients with stable coronary artery disease [8].
6. perform a hyperemic transient simulation prescribing at inlet a a properly scaled aortic pressure waveform (normalized curve is reported in Fig. 4) and coupling at outlets lumped parameter models with the resistances previously computed (Scenario II);
7. use results from simulation performed in Step 6 to estimate FFR.

**Fig. 3:**
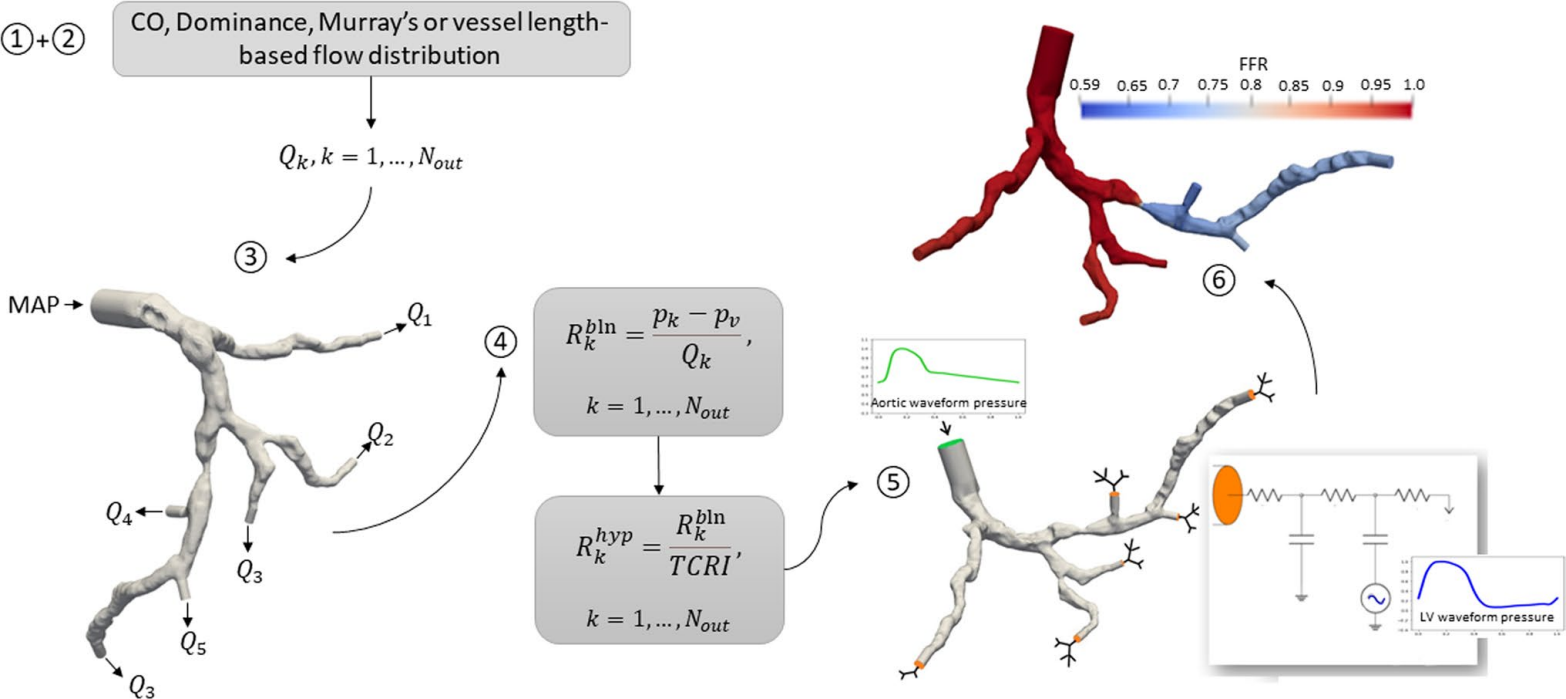
Illustration of the modelling for FFR prediction: (1) + (2) definition of total coronary flow and its distribution among outlets, (3) baseline simulation, (4) compute peripheral coronary resistances based on resting simulation’s results, (5) hyperemic simulation, (6) extraction of computational FFR

There is a variety of methods used in the literature to distribute baseline coronary flow among the coronary vessels [13]. We decided to use two different methods, distal Murray flow distribution and a vessel length-based flow distribution, in order to have more simulations with different flows. Distal Murray method assumes a proportionality between the flow and the cube of the outlets’ vessel diameters [26]. Although a modified rule $q\sim d^{2.66}$ has be proven to better fit flow distribution in coronary arteries [27], in the adopted pipeline Murray’s exponent is set to 3 according to the original work. However, the study reported in [13] highlights that the sensitivity of FFR prediction to variations of Murray’s exponent assumed to be a uniform variable with values between 2.0 and 3.0 is extremely low and certainly orders of magnitude lower than sensitivity to other parameters used in the modelling pipeline. On the other hand, in the vessel length-based method the flow is distributed among all outlets using a stem-and-crown model, which is based on allometric scaling between the length of coronary arterial tree and the myocardial mass [28, 29]. With Murray’s flow setup we refer to hyperemic simulation setting in which peripheral coronary resistances are extracted from baseline simulation’s results with the flow distributed according Murray’s law, while with vessel length-based flow setup we refer to hyperemic simulation setting in which peripheral coronary resistances are extracted from baseline simulation’s results with the flow distributed according the vessel length-based method. See Fig. 3 for an overview of the FFR-prediction pipeline.

### Patient-Specific Parameters

The parameters required to perform baseline and hyperemic simulations were extracted from patient-specific clinical data.

For steady state baseline simulations, the mean arterial pressure (MAP) computed as a linear combination of diastolic and systolic blood pressure was prescribed at the inlet of the computational domain, while the total baseline flow to distribute among outlets was assumed to be a portion of the measured CO: $q_{cor} = 0.045$ CO [30] and then distributed according to the selected flow distribution methods.

**Fig. 4:**
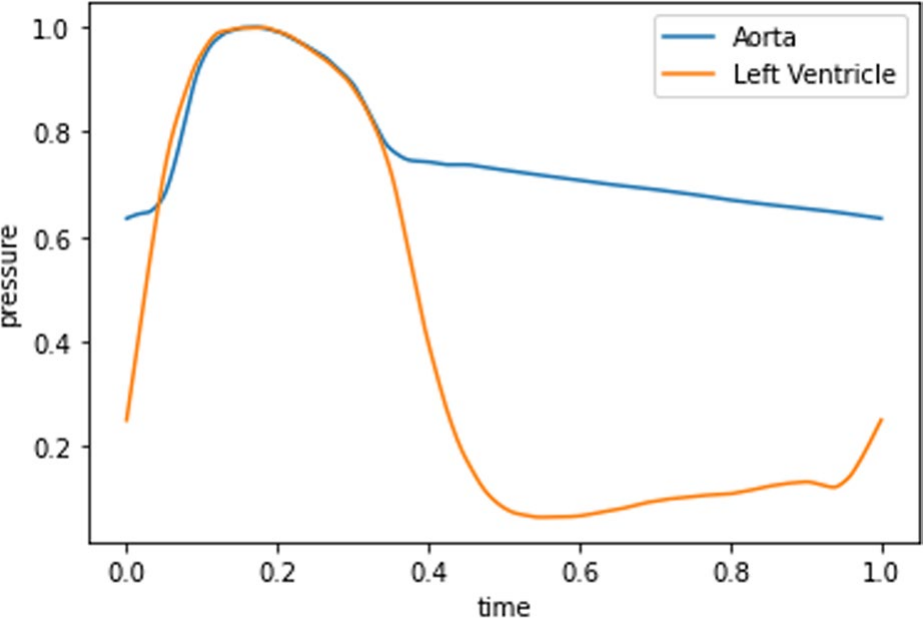
Aortic and left ventricle characteristic waveforms used for patient-specific simulations. Pressure and time are normalized values. The waveform shape are taken from [35]

For transient hyperemic simulations, MAP, pulse pressure and cardiac cycle duration, extracted from clinical tracings, were used to scale the prescribed aortic and left ventricle pressure waveforms, at the inlet section and in lumped parameter models coupled at outlets, respectively. These pressures were not directly measured, rather approximated via an assumed normalized curve scaled by the patient-specific data. Changing the shape of these curves should not affect FFR estimations significantly. This is due to the fact that FFR is based on the ratio of cardiac cycle-averaged pressures, and that, for this particular context of coronary blood flow, transient aspects do not represent a significant part of cardiac cycle-averaged pressure drops. Indeed, several studies [8, 31] have shown that FFR predicted with steady state simulations reproduce very well FFR estimations performed with transient simulations. Normalized aortic and left ventricle characteristic waveforms used for patient-specific simulations are shown in Fig. 4. Total peripheral compliance was computed as a portion of the arterial compliance of 1.7 mL mmHg^-1^ and then distributed among outlets according to Murray’s law. This modelling choice is related to the fact that peripheral vascular compliance distribution is assumed to be directly proportional to flow distribution, as adopted in many modelling works [32–34]. The resulting $C_{out,k}$ and $R_{out,k}^{hyp}$ computed in (2) have to be subsequently distributed among the three different compartments of the Windkessel model coupled to the k-th outlet. The fractions for distributing $R_{out,k}^{hyp}$ among $R_{a}$, $R_{a-micro}$, $R_{d}$, in relation to Fig. 2, are 0.01, 0.84, 0.15, respectively. In the same way $C_{out,k}$ is distributed among $C_{a}$ and $C_{myo}$ according to fraction 0.025 and 0.975. Parameter distribution among components of lumped-parameter models was taken from [8].

## Results

The effects of guidewire insertion on coronary hemodynamics are studied and presented for a population of 18 patients. The 24 FFR measurements collected are distributed among four different ranges of values: 0.38$-$0.52, 0.52$-$0.72, 0.72$-$0.84, 0.84-1. Each FFR range defines a class, we refer to class 1 as the group of the most severe stenoses, while class 4 represents the group of the less severe stenoses.

The results are presented in terms of reduction in mean coronary hyperemic flow rate, difference in pressure drop and resulting effect on FFR. The predicted FFR are then evaluated against the invasive FFR clinically measured.

**Fig. 5:**
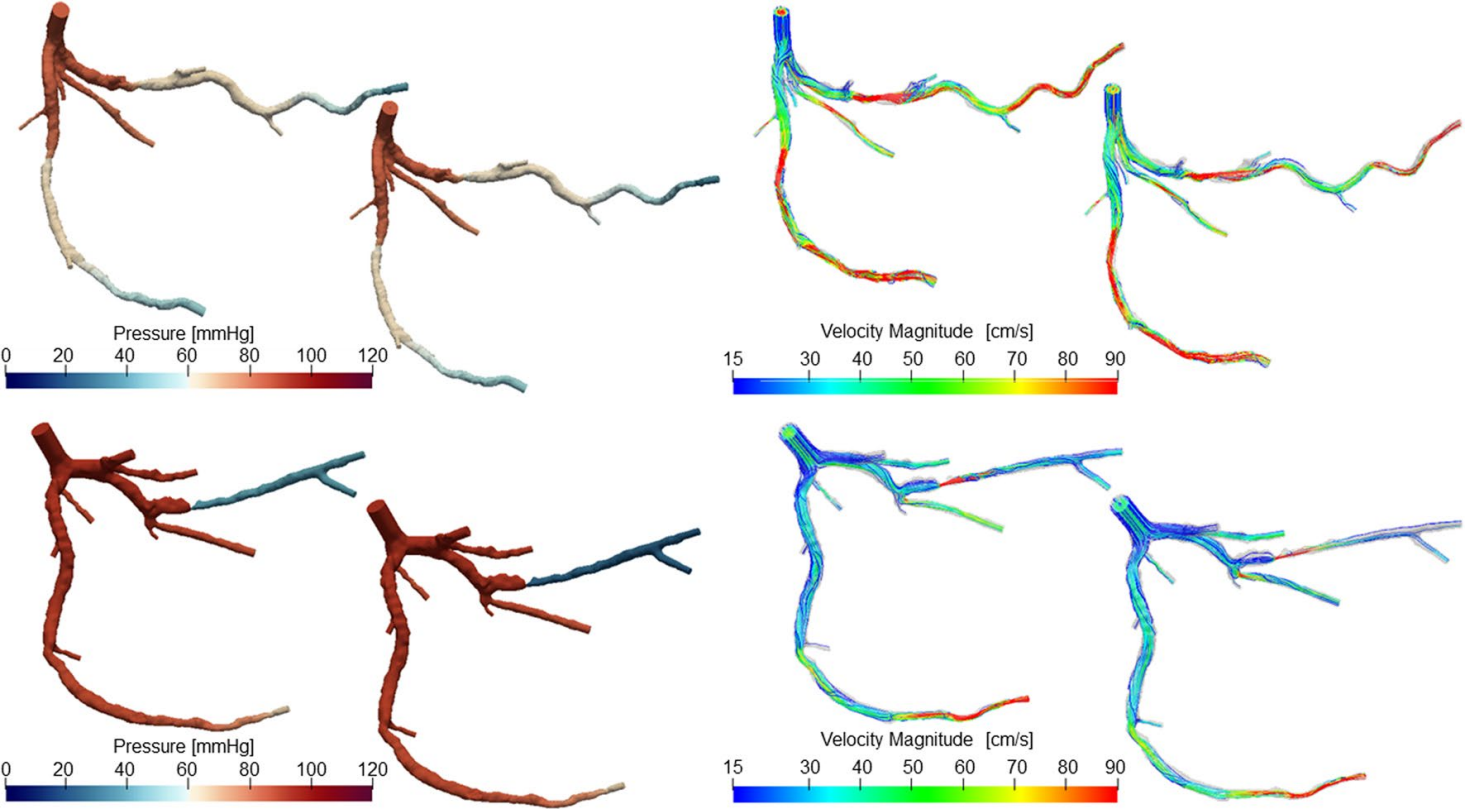
Pressure distribution (left) and velocity field (right) in the wire-absent configuration (domain on the left in each panel) and then in the wire-included configuration (domain on the right in each panel). At the top are reported results for Patient 27, while Patient 15 is represented at the bottom. In both cases the flow has been distributed according to the vessel length-based flow setup

Moreover the resulting pressure distribution and velocity field for some patients are shown in Fig. 5.

### Guidewire Insertion Effect on Flow Discharge

To observe the guidewire flow-obstruction effects, the pulsatile flow rate has been calculated at the inlet section in both conditions: with and without insertion of guidewire into the stenotic branch of each coronary tree. The presence of the pressure guidewire is reflected in a decrease of the mean total flow rate respect to the condition without the guidewire for both flow distribution setups and for all FFR classes. In particular, the average flow reduction observed after the inclusion of the guidewire is of $4.58\%$ for Murray’s flow setup and of $6.98\%$ for the vessel length-based flow setup. Average values for each FFR classes are reported in Table 3.

### Guidewire Insertion Effect on Pressure

We computed the trans-stenotic pressure drop after the lesion for both conditions. We observed that a reduction of the lumen contributes to increase the pressure drop and the insertion of the pressure guidewire in the simulations enhances this drop. In the wire-included configuration we noted an average increase of pressure drop of $42.23\%$ across all FFR classes in the Murray’s flow setup and of $18.93\%$ using the vessel length-based flow setup.

### Guidewire Insertion Effect of FFR Measurements

**Fig. 6:**
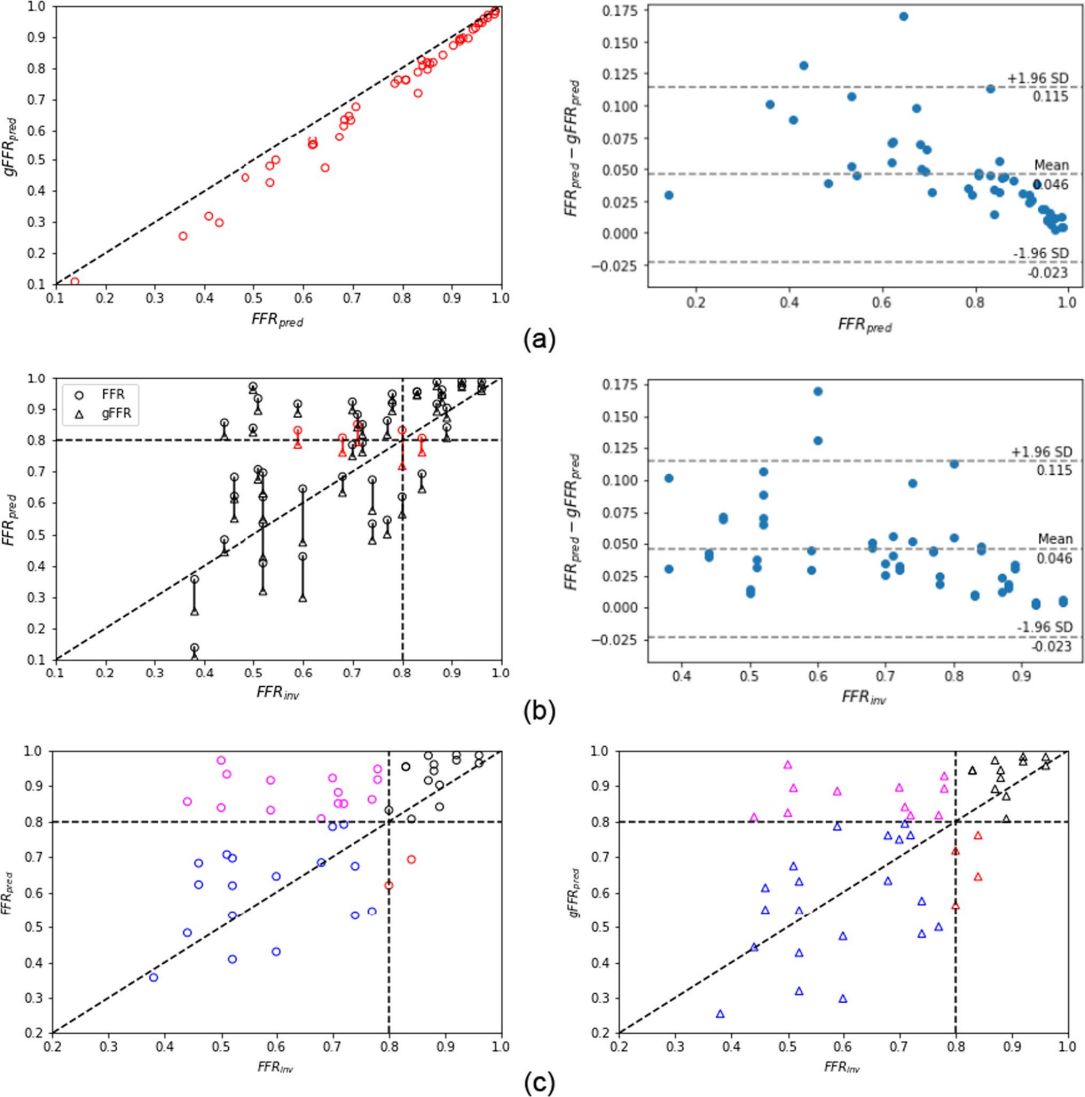
FFR predictions obtained based on Murray’s flow distribution. **a** Comparison of FFR_pred_ and gFFR_pred_. Scatter plot (left) and Bland-Altman plot (right). **b** Comparison of predicted FFR in both models, wire-absent and wire-included model, and invasive FFR_inv_. Scatter plot (left) and Bland-Altman plot (right). The scatter plot also highlights (in red) cases which have different classification (FFR $\le$ 0.8) depending on having the guidewire present or not. **c** Comparison of FFR_pred_ (left) and gFFR_pred_ (right) against FFR_inv_. The horizontal and vertical dashed lines represent the FFR cut-off value for classifying ischemia causing stenoses. False negative results are highlighted in magenta, false positive results are colored in red, while results correctly identified are reported in blue in case of true positive or in black in case of true negative

**Fig. 7:**
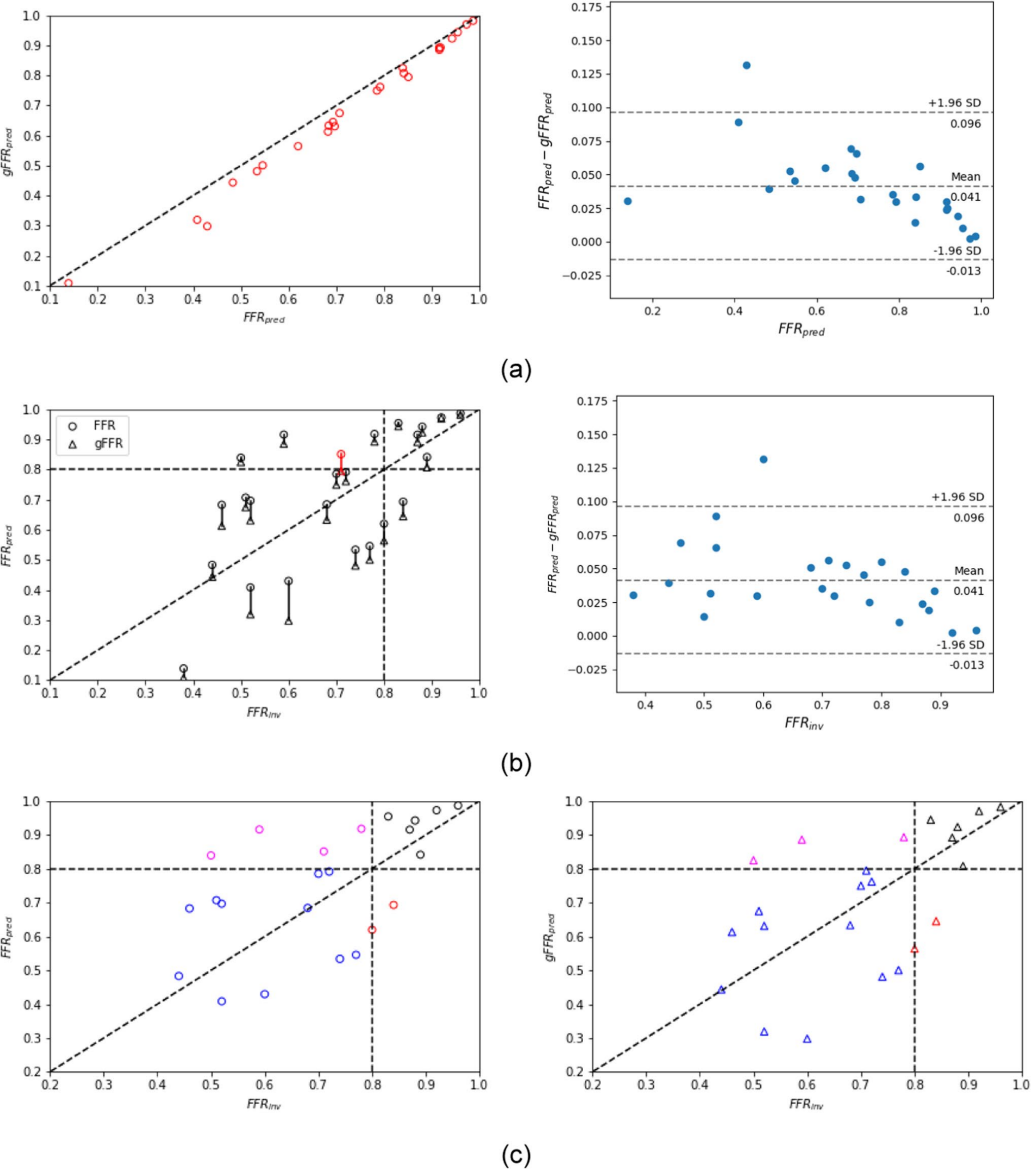
FFR prediction obtained based on the vessel length-based flow distribution. **a** Comparison of FFR_pred_ and gFFR_pred_. Scatter plot (left) and Bland-Altman plot (right). **b** Comparison of predicted FFR in both models, wire-absent and wire-included model, and invasive FFR_inv_. Scatter plot (left) and Bland-Altman plot (right). The scatter plot also highlights (in red) cases which have different classification (FFR $\le$ 0.8) depending on having the guidewire present or not. **c** Comparison of FFR_pred_ (left) and gFFR_pred_ (right) against FFR_inv_. The horizontal and vertical dashed lines represent the FFR cut-off value for classifying ischemia causing stenoses. False negative results are highlighted in magenta, false positive results are colored in red, while results correctly identified are reported in blue in case of true positive or in black in case of true negative

FFR is calculated as the ratio of pressure distal to the stenosis $p_d$ computed at the same location where it was clinically measured, to the pressure computed at the ostium of the coronary tree $p_a$. $p_d$ and $p_a$ are obtained as average values of the cross-sectional pressure over one cardiac cycle. We refer to FFR_pred_ as the computational FFR predicted using the wire-absent condition, while to gFFR_pred_ as the computational FFR predicted using the wire-included condition. Figures 6a and 7a compare gFFR_pred_ against FFR_pred_ and show the FFR_pred_-gFFR_pred_ characteristics for all stenoses under pulsatile hyperemic flow for each flow distribution setups respectively. The difference between FFR_pred_ and gFFR_pred_ increases as the stenosis severity increases. We observed that in the Murray’s flow setup the value of predicted gFFR_pred_ decreases on average by $2.2\%$, $6.7\%$, $9.4\%$, $11.7\%$ for class 4, class 3, class 2, class1, respectively. The same trend is recorded also for the predicted gFFR_pred_ with the vessel length-based flow setup. In this configuration, $2.7\%$, $5.3\%$, $10.7\%$, $11.4\%$ are the decrease in FFR for the four ordered class after the inclusion of the wire. The average values are reported in Table 3. Figures 6b and 7b compare gFFR_pred_ and FFR_pred_ against the invasive FFR and show the FFR_pred_-gFFR_pred_ characteristics for all stenoses under pulsatile hyperemic flow for each flow distribution setups respectively. A numerical characterization of the comparison is given in Table 4.

**Table 3:** For each FFR class here considered we report the mean percentage of predicted FFR drop, increase of trans-stenotic pressure drop and decrease of hyperemic flow rate which we recorded when we move from the wire-absent model to the wire-included model

	Murray’s flow setup		
FFR class	Drop in FFR (%)	Rise in $\Delta$P (%)	Drop in inflow (%)
Class 4	2.18	39.06	3.21
Class 3	6.70	44.34	5.94
Class 2	9.40	47.50	4.47
Class 1	11.70	38.00	4.69

	Vessel length-based flow setup		
FFR class	Drop in FFR (%)	Rise in $\Delta$P (%)	Drop in inflow (%)
Class 4	2.71	19.00	5.56
Class 3	5.3	21.84	5.48
Class 2	10.7	19.64	9.94
Class 1	11.3	15.24	6.94

## Discussion

In this study we have adopted the modelling framework for FFR prediction proposed and validated in [8] and [13] in order to assess the impact of the presence of the pressure guidewire in FFR prediction.

Analysing and comparing the results, we can say that the introduction of the pressure guidewire in CFD simulations can play a significant role affecting the pressure and flow predictions, especially in case of intermediate and severe stenoses. Its presence is associated with an additional pressure loss and a decrease of flow rate, as reported in Table 3. These haemodynamic changes affect the prediction of FFR leading to a tendency of predicting a lower FFR value. We have observed that the FFR reduction depends on the severity of the stenosis, as it is shown in Figs. 6a and 7b. Indeed, the difference between the FFR_pred_ and the gFFR_pred_ is less prominent in cases of moderate stenosis, while it is major in more severe cases, where a drop of 11.5% is on averaged recorded compared to an averaged reduction of 2.4% in less severe disease status. In addition, by comparing the results from the two different flow distributions applied in this study and taking into account the findings reported in [13] and in [36], we observe that the impact of the wire on FFR seems to be smaller than sensitivity reported for other factors such as flow distribution technique, total flow and uncertainty in coronary tree segmentation. However, although FFR is very sensitive to the flow distribution, we still see a significant impact from the presence of the guidewire, regardless of which flow distribution was used. Rather, the impact of the catheter shows a dependence with respect to the degree of disease severity.

On the other hand, from a clinical decision-making point of view different conclusions can be drawn. For stenoses with associated FFR included in class 4, class 2 or class 1 the impact of the pressure guidewire on stenosis evaluation and clinical decision is of less significance. Indeed, in the first case, the guidewire has negligible effects on predicted FFR, while, on the other hand, the presence of the wire worsens a clinical situation already severe in which the therapy to follow is already evident. The situation is different for intermediate stenoses associated with a FFR included in class 3. In this case the presence of the pressure guidewire could change the diagnosis and a predicted FFR indicating a non-significant ischemia could drop to the “gray zone” of clinical uncertainty or even suggest a need of surgical intervention when the wire is included in the model, as it is shown in the left plots of Figs. 6b and 7b. Therefore, the results in this preliminary study indicate that from a clinical standpoint, the inclusion of the catheter is only relevant in predicting FFR for intermediate stenoses.

**Table 4:** Diagnostic index of wire-absent and wire-included model for both flow distribution setup

	Murray’s flow setup	
	Wire-absent model	Wire-included model
Sensitivity	0.35	0.53
Specificity	1.00	0.86
Accuracy	0.54	0.62
Standard deviation	0.14	0.16
Bias	0.13	0.08
AUC	0.84	0.86

	Vessel length-based flow setup	
	Wire-absent model	Wire-included model
Sensitivity	0.76	0.82
Specificity	0.86	0.86
Accuracy	0.79	0.83
Standard deviation	0.17	0.18
Bias	0.03	− 0.01
AUC	0.88	0.90

Sensitivity, specificity and accuracy are described in terms of true positive (TP), true negative (TN), false negative (FN) and false positive (FP) as Sensitivity = TP/(TP+FN), Specificity = TN/(TN+FP), Accuracy = (TN+TP)/(TN+TP+FN+FP). The AUC presents the area under the Receiver Operating Characteristics (ROC) curve plotted by using true positive rate against false positive rate for different cut-points of the diagnostic test

Table 4 reports the statistics that describe the efficiency of our models as diagnostic tests, while in Figs. 6c and 7c we have their qualitative representation highlighting with different colors predictions resulting in a correct classification (blue and black) and false classification (magenta and red) for both configurations considered in this study. We observe that the inclusion of the guidewire in the model leads to a overall improvement of the diagnostic capability of the model balancing accuracy, specificity and sensitivity. In addition, the bias from the clinical measured FFR drops from 0.13 to 0.08 in Murray’s law setup and from 0.03 to $-$0.01 in vessel length-based flow setup when the physical presence of the pressure guidewire is taken into account.

In conclusion, our results show that the impact of accounting for the presence of the pressure guidewire in model-based FFR prediction pipelines can play a relevant role, since the catheter affects pressure and flow measurements, especially for intermediate and severe stenoses. However, the impact of the pressure guidewire has a different clinical relevance depending on stenosis severity, as it results to be especially influential on clinical decisions only in cases of intermediate stenosis. Including this feature in a modelling pipeline would allow to reduce modelling errors by more reliably representing the clinical setting in which FFR measurements are performed. Nevertheless, an improvement of agreement between clinical and predicted FFR is not necessarily to be expected. Errors related to modeling uncertainties in the boundary conditions and geometry have a bigger impact on predictions.

### Limitations

The pressure guidewire is assumed to be rigid and static placed axially along the stenotic branch. This represents an ideal situation that not always can be produced in practice since the wire is susceptible to unsteady blood flow. Effects of different positions of the guidewire inside the vessel and a its interaction with the fluid should be investigated. Some modelling hypotheses should be explored. As emerged in the study conducted in [13], the most important parameters in terms of sensitivity and uncertainty of FFR prediction concise with the hyperemic factor and the definition of baseline flow through the coronary tree. Hence, the impact of patient-specific TCRI, that accounts for lesion-induced reduced vasodilatory capacity during adenosine administration, on FFR prediction should be addressed, as well as different quantification methods of total baseline coronary flow should be investigated. Another limitations is provided by the rigid wall assumption. Although for our application such an assumption could be considered acceptable since the main focus of this work is to compare two scenarios (with and without guide) both affected by the same limitation, the use of a fluid–structure interaction model could improve the accuracy of the predicted FFR with respect to the invasive one. Finally, we have limited the analysis to six FFR measurements for each of the four classes. A larger patient population should be considered in order to include a wider range of disease states, making sure to comprise a sufficient number of intermediate stenoses patients.
